# TOPICAL TREATMENT OF MELASMA

**DOI:** 10.4103/0019-5154.57602

**Published:** 2009

**Authors:** Debabrata Bandyopadhyay

**Affiliations:** *From the Department of Dermatology, Venereology, and Leprosy, R. G. Kar Medical College, Kolkata, India.*

**Keywords:** *Melasma*, *newer agents*, *topical treatment*

## Abstract

Melasma is a common hypermelanotic disorder affecting the face that is associated with considerable psychological impacts. The management of melasma is challenging and requires a long-term treatment plan. In addition to avoidance of aggravating factors like oral pills and ultraviolet exposure, topical therapy has remained the mainstay of treatment. Multiple options for topical treatment are available, of which hydroquinone (HQ) is the most commonly prescribed agent. Besides HQ, other topical agents for which varying degrees of evidence for clinical efficacy exist include azelaic acid, kojic acid, retinoids, topical steroids, glycolic acid, mequinol, and arbutin. Topical medications modify various stages of melanogenesis, the most common mode of action being inhibition of the enzyme, tyrosinase. Combination therapy is the preferred mode of treatment for the synergism and reduction of untoward effects. The most popular combination consists of HQ, a topical steroid, and retinoic acid. Prolonged HQ usage may lead to untoward effects like depigmentation and exogenous ochronosis. The search for safer alternatives has given rise to the development of many newer agents, several of them from natural sources. Well-designed controlled clinical trials are needed to clarify their role in the routine management of melasma.

## Introduction

Melasma (from the Greek word, ‘*melas*’ meaning black) is a common, acquired, circumscribed hypermelanosis of sun-exposed skin. It presents as symmetric, hyperpigmented macules having irregular, serrated, and geographic borders. The most common locations are the cheeks, upper lips, the chin, and the forehead, but other sun-exposed areas may also occasionally be involved. The term, “chloasma” (from the Greek word, ‘*chloazein*’ meaning ‘to be green’) is often used to describe melasma developing during pregnancy; however, as the pigmentation never appears to be green, the term, “melasma” should be preferred.

Although melasma may affect any race, it is much more common in constitutionally darker skin types (skin types IV to VI) than in lighter skin types, and it may be more common in light brown skins, especially in people of East Asian, Southeast Asian, and Hispanic origin who live in areas of the world with intense solar ultraviolet exposure. Melasma is the most common pigmentary disorder among Indians.[1]. It is much more common in women during their reproductive years but about 10% of the cases do occur in men. The clinical and histological features of melasma in men are the same as those of melasma in women.[2]

## Pathophysiology of Melasma

The pathophysiology of melasma remains elusive, but multiple factors have been implicated. The role of female hormonal activity has been suggested by the increased frequency of occurrence of melasma in pregnancy and in those on oral contraceptive pills, estrogen replacement therapy, and estrogen treatment for prostatic cancer. The mechanism of induction of melasma by estrogen may be related to the presence of estrogen receptors on the melanocytes that stimulate the cells to produce more melanin. Genetic factors are indicated by familial occurrence of melasma and its increased incidence in people of Asian and Hispanic origins. Other factors implicated in the etiopathogenesis of melasma are photosensitizing and anticonvulsant medications, mild ovarian or thyroid dysfunction, and certain cosmetics. One of the most important factors in the development of melasma is ultraviolet exposure from sunlight or other sources. Exacerbation of melasma is universally seen after prolonged sunexposure but the pigmentation fades after periods of avoidance of sunexposure. Whatever the mechanisms, melasma results in an increased deposition of melanin in the epidermis, in the dermis within melanophages, or both. The number of melanocytes in the lesions has been variably reported to be normal[3] or increased.[4] The melanosomes within the melanocytes and keratinocytes have been reported to be increased in size.[3,4]

## Types of Melasma

The lesions range in color from light brown to dark brownish-black and affect the regions of the face in different patterns. Three clinical patterns of distribution of the pigmentation may be recognized: Centrofacial, malar, and mandibular.[5]

The *centrofacial pattern* is the most common and involves the cheeks, nose, forehead, upper lip, and chin. The *malar pattern* involves the cheeks and nose. The ramus of the mandible is involved in the *mandibular pattern*. Melasma does not involve the mucous membrane.

With the help of Wood's lamp examination, melasma may be classified into four histological types according to the depth of pigment deposition[6]. The *epidermal type* is the most common in which the pigmentation appears more intense under Wood's lamp examination. Melanin is distributed throughout the epidermis; topical treatment may work best in this type of melasma. In the *dermal type*, the pigmentation is not intensified with Wood's light. The pigmentation is due to plenty of melanophages in the dermis. In the *mixed type*, Wood's light intensifies pigmentation in some areas while other areas remain unchanged. The pigmentation is due to increased epidermal melanin as well as dermal melanophages. Wood's lamp examination is of no benefit in very dark individuals, and this type is classified as *indeterminate*. This classification may partly work in lighter skin types but not in brown or black skin types.[7] Moreover, there may not be good correlation between the findings of Wood's lamp examination and histological depth of pigmentation.[7]

Depending on the natural history of the lesions, melasma may also be classified into *transient* and *persistent* types.[7] The transient type disappears within one year of cessation of hormonal stimuli like pregnancy or oral contraceptive pills. The persistent type continues to be present more than one year after the hormonal stimulus is removed and is caused by the action of UV rays and other factors, highlighting the role of sun-avoidance in the management of melasma.

## Topical Treatment

By causing cosmetic disfigurement of the face, melasma is frequently associated with a significant emotional effect. There is no universally effective specific therapy for the disease—existing agents have varying degrees of effectiveness, and the condition, more often than not, relapses.[8] Most cases are treated with topical agents, used alone, or in combinations. Other modalities of treatment utilized in the management of this hypermelanotic disorder are chemical peels and physical therapies in the form of various lasers or intense pulse light sources. All patients with melasma should be counseled about the natural course of the disease and the necessity for adherence to a long-term treatment plan. Careful history about the possible precipitating or aggravating factors must be taken with special attention to the intake of oral contraceptives or other hormonal preparations, phototoxic and anti-seizure medications, and the usage of cosmetics. Discontinuation of oral pills and avoidance of scented cosmetics is advised. Recurrence of melasma occurs on exposure to sunlight and other sources of ultraviolet rays. Photoprotective measures like the avoidance of direct sun-exposure and the regular use of a broad-spectrum sunscreen are always advised, although clinical studies on their role are lacking. Treatment with demelanizing agents must be continued for several months before significant clinical benefits become noticeable. Topical agents are much more effective in the epidermal type of melasma.[9]

### Hydroquinone

Hydroquinone (HQ), also known as dihydroxybenzene, is a hydroxyphenolic compound that is structurally similar to precursors of melanin. It inhibits the conversion of DOPA to melanin by inhibition of the enzyme, tyrosinase. HQ affects not only the formation, melanization, and degradation of melanosomes, but it also affects the membranous structures of melanocytes and eventually causes necrosis of whole melanocytes.[10] HQ is an oxidizing agent that can oxidize in tubes or bottles, turning the color of formulations from white to brown. Products that have undergone this color change are ineffective and should be discarded.[11]

HQ is the most frequently prescribed depigmenting agent worldwide and it has remained the gold standard for the treatment of melasma, particularly of the epidermal type. HQ preparations are commonly used in the treatment of melasma at concentrations varying from 2 to 5% applied once daily. Variably good yet reversible results are obtained in most of the patients treated with HQ. The depigmenting effects of the HQ treatment become evident after 5-7 weeks. Treatment should be continued for at least three months, up to one year.[9] HQ is also formulated in combination with other agents like sunscreens, topical steroids, retinoids, and glycolic acids for added benefits.

Adverse reactions of HQ are related to its dose and the duration of treatment. Irritation is the most common complication; other adverse effects include erythema, stinging, colloid milium, irritant and allergic contact dermatitis, nail discoloration, transient hypochromia, and paradoxical postinflammatory hypermelanosis.[9,12] The so-called ‘confetti-like’ depigmentation or guttate hypomelanosis is characterized by mottled depigmented spots that develop on the macules of melasma. This is observed in association with the use of HQ at concentrations higher than 2%. Exogenous ochronosis[13,14] a blue-black pigmentation of the treated areas, has been mainly reported in darker skin from prolonged use of a strong concentration, but has also been reported in whites after short- or long-term use of 2% HQ preparations.[15] The etiology of hydroquinone-induced hyperpigmentation in exogenous ochronosis remains speculative; improvement occurs very slowly after avoidance of the offending agent.[16] The monobenzy lether of HQ should never be used in the treatment of melasma as it can produce a permanent loss of melanocytes, causing a disfiguring confetti-like leukoderma.

Regulatory agencies in Japan, Europe, and most recently the USA, have raised questions about the safety of HQ[17] and it has been banned in cosmetic preparations in many countries. This has encouraged research into alternative agents for the topical management of melasma.

### Azelaic acid

Azelaic acid is a naturally occurring, nonphenolic, saturated, nine-carbon dicarboxylic acid that competitively inhibits tyrosinase. Azelaic acid was initially developed as a topical anti-acne agent but because of its effect on tyrosinase, it has also been used to treat hyperpigmentary disorders like melasma. Its mechanisms of action include the inhibition of DNA synthesis and mitochondrial enzymes, thereby inducing direct cytotoxic effects toward the melanocyte.[18] Topical azelaic acid has no depigmentation effect on normally pigmented skin; this specificity may be attributed to its selective effects on abnormal melanocytes.[19] It can be used for postinflammatory hyperpigmentation in acne. Free radicals are believed to contribute to hyperpigmentation, and azelaic acid acts by reducing free radical production.[20]

A double-blind randomized study has shown that a 20% concentration of azelaic acid was equivalent to 4% hydroquinone in the treatment of melasma, but without its side effects.[21] Another controlled study has found azelaic acid to be superior to 2% hydroquinone.[22] A combination of azelaic acid with 0.05% tretinoin or 15-20% glycolic acid may produce earlier, more pronounced skin lightening. Adverse effects of azelaic acid include pruritus, mild erythema, and burning.[23]

### Kojic acid

Kojic acid (5-hydroxy-2-hydroxymethyl-4-pyrone) is a naturally occurring, hydrophilic fungal product derived from certain species of *Acetobacter*, *Aspergillus*, and *Penicillium*.[19] It acts by inhibiting the production of free tyrosinase; it is also a potent antioxidant.[24] It is generally univalent to other therapies but may be more irritating. Kojic (KA) acid is used at concentrations ranging from 1 to 4%. In one double-blind study, KA 2% combined with HQ 2% was shown to be superior to glycolic acid (GA) 10% and HQ 2%[25] Another double-blind study compared GA 5% with either HQ 4% or KA 4% for three months. Both combinations proved equally effective with a reduction of pigmentation in 52% of the patients.[26] KA may be effective if a patient has difficulty tolerating other first-line therapies. It may cause contact dermatitis and erythema.[27]

### Retinoids

Retinoids, such as tretinoin, were first used in combination with HQ as penetration enhancers, but were later recognized to have their own effect on melanogenesis.[28] Retinoids affect multiple steps in the melanization pathway. Tretinoin promotes the rapid loss of pigment through epidermopoiesis and increased epidermal turnover decreases the contact time between keratinocytes and melanocytes.[8] Retinoic acid (RA) suppresses UVB-induced pigmentation by reducing tyrosinase activity. The acid acts at a posttranscriptional level on tyrosinase and tyrosinase-related protein.[12] Compared with phenolic compounds like HQ, RA takes a much longer time to act; clinically significant lightening becomes.evident after 24 weeks.

Tretinoin monotherapy has produced a good therapeutic response in clinical trials[29,30] but better results are obtained in combination with other agents like HQ and corticosteroids. The most common side effects include erythema, burning, stinging, dryness, and scaling. The inflammation may cause hyperpigmentation, particularly in people with dark skin.[28] Patients must be advised to use sunscreens during treatment with retinoic acid. Adapalene, a naphthoic acid derivative with retinoid activity, was found to be equally efficacious in a randomized trial in Indian patients but with significantly less untoward effects than tretinoin.[31]

### Topical steroids

Reversible hypopigmentation of normal skin is a well-known untoward effect of prolonged potent steroid application. The mechanism of the skin-lightening effect of topical corticosteroids is ill-understood. Melanocytes respond to a wide variety of chemical mediators. The inhibitory effects of corticosteroids on the synthesis of mediators like prostaglandin and leukotriene may partly explain their effects on melanogenesis.[28] Potent or superpotent steroids, when used alone, have been associated with good therapeutic responses,[32,33] but monotherapy is not recommended due to their frequent untoward effects.

Topical steroids are used in combination products for their synergistic effects and for the reduction of irritation from other products like tretinoin. Various combinations with HQ and retinoic acid have given good cosmetic results in clinical trials.[34] Adverse effects of topical steroids include irritation, rosacea-like dermatosis, atrophy, telangiectasia, and hypertrichosis.

### Glycolic acid

Glycolic acid is an alpha-hydroxy acid that is usually combined with other agents at a concentration of 5-10% for its skin-lightening property. The mechanism of its effect might be due to epidermal remodeling and accelerated desquamation, which would result in quick pigment dispersion on pigmentary lesions. It also directly reduces melanin formation in melanocytes by tyrosinase inhibition.[35]

A randomized controlled trial has demonstrated that a formulation containing 10% glycolic acid and 4% HQ had good clinical efficacy in treating melasma in a group of Hispanic patients. Irritation was a common side effect which resolved with the temporary cessation of application and application of moisturizers.[36]

### Mequinol

Mequinol (4-hydroxyanisole, hydroquinone monomethyl ether) is a derivative of HQ. Its mechanism of action is unclear; however, being a substrate of tyrosinase, it may act as a competitive inhibitor of the formation of melanin precursors[11]. It is currently marketed in the USA at a concentration of 2% in combination with 0.01% tretinoin as a penetration enhancer. In a randomized, parallel-group, double-masked study involving 216 subjects, a mequinol 2%/ tretinoin 0.01% solution was found to be highly effective and well-tolerated treatment for solar lentigines and related hyperpigmented lesions, being superior to HQ 3% for lesions on the forearm and of similar efficacy for lesions on the face.[37] The results of a trial with a case series of male patients with melasma has shown that four out of five patients achieved complete clearance at 12 weeks, and one patient showed moderate improvement. Side effects were minimal and consisted of stinging in one patient. All patients maintained results at the 16-weeks' follow-up visit.[38]

An open-label study was conducted to determine the rate of adverse events of topical mequinol/tretinoin, twice daily application for up to 24 weeks with concomitant sunscreen in the treatment of solar lentigines and related hyperpigmented lesions. The untoward effects of this topical combination were in the form of erythema, burning/stinging/tingling, desquamation, pruritus, skin irritation, halo hypopigmentation, and hypopigmentation. The authors concluded that when used with sunscreen of SPF 25 or greater, the combination was safe and well tolerated and did not produce any unexpected or unusual adverse events.[39] Controlled clinical trials with large numbers of patients are needed to clarify the role of this promising new agent in the routine management of melasma.

### Arbutin

Arbutin, the *beta*-D-glucopyranoside derivative of hydroquinone, is a naturally occurring plant product which has been used successfully in the treatment of hyperpigmentary disorders. The glycosidic bond is hydrolyzed *in vivo* leading to the controlled release of hydroquinone.[12] Arbutin acts by the inhibition of tyrosinase, thereby decreasing melanin formation. The normal skin microflora may also hydrolyze arbutin; the hydrolyzed hydroquinone shows more potent free-radical scavenging activity and tyrosinase inhibition than arbutin.[40]

The action of arbutin is dose-dependent and less toxic than hydroquinone. *Deoxyarbutin* is a recently developed derivative of arbutin that has been produced by removing the hydroxyl groups from the molecule. This produces reversible skin-lightening by direct inhibition of tyrosinase.[41] Although good controlled clinical trials are lacking, initial *in vitro* and *in vivo* experimental studies have demonstrated that it could be a safe and effective treatment for hypermelanotic disorders.[42,43]

## Other New and Experimental Agents

A number of agents, both synthetic and those derived from natural sources like plants, have been investigated for their potential role in reducing melanin pigmentation. Although experimental evidence suggests their possible benefits, dependable controlled clinical trials are mostly lacking. Some of the compounds are formulated in combination products and marketed by pharmaceutical companies; many are available as ingredients of over-the-counter preparations.

### N-acetyl-4-S-cysteaminylphenol

NCAP is a phenolic agent which acts as an alternative substrate for tyrosinase, thus inhibiting the enzyme's activity. It has been reported to be more stable and causes less irritation than hydroquinone. In a study of 12 patients with melasma, using 4% NCAP, 66% of patients showed marked improvement and 8% showed a complete resolution of melasma lesions. With daily topical application, clinical changes were evident after 2-4 weeks.[44]

## Alpha-tocopheryl Ferulate

The compound of alpha-tocopherol and ferulic acid, also an antioxidant connected with an ester bond, alpha-tocopheryl ferulate (alpha-TF), can absorb ultraviolet (UV) radiation and thus maintain tocopherol in a stable state. In experimental studies, this agent was found to have significant effect in the retardation of melanogenesis, possibly by inhibiting tyrosine hydroxylase activity in an indirect manner.[45,46]

### Ascorbic acid

Ascorbic acid has antioxidant properties and affects melanogenesis by reducing dopaquinone to DOPA and preventing free-radical production and absorption of ultraviolet radiation.[12] Comparing the efficacy of 5% ascorbic acid and 4% hydroquinone in 16 patients with melasma in a double-blind clinical trial, the authors concluded that although hydroquinone showed a better response, ascorbic acid may play a role in the therapy of melasma as it is almost devoid of side effects and it could be used alone or in combination therapy.[47] In an open-label trial, 25% L-ascorbic acid formulated with a penetration enhancer, was found to have a significant effect in the treatment of melasma.[48] Ascorbic acid, however, is highly unstable in aqueous solution and stable esters like *magnesium ascorbyl-2-phosphate* (MAP) have been synthesized. MAP has a protective effect against UVB radiation[49] and it inhibits melanogenesis *in vitro* and *in vivo*. Experiments demonstrated that MAP cream was absorbed into the epidermis and that 1.6% remained 48 hours after application. The lightening effect was significant in 19 of 34 patients with melasma or senile freckles and in 3 of 25 patients with normal skin.[50]

### Niacinamide

Niacinamide (nicotinamide), the biologically active amide form of niacin (vitamin B_3_), can reduce pigmentation by reversibly preventing the transfer of melanosomes from melanocytes to the keratinocytes.[51] It has no effect on tyrosinase activity. In clinical studies, niacinamide significantly decreased hyperpigmentation and increased skin lightness compared with vehicle alone after four weeks of use.[52]

### Liquorice derivatives

Liquorice is the root of the perennial herb Glycyrrhiza glabra. *Glabridin* is an oil-soluble derivative of liquorice extract. *Glabridin* has been shown to have tyrosinase inhibitory as well as anti-inflammatory properties in experimental studies.[53] A clinical trial with *Liquiritin*, another liquorice derivative, has also shown benefit in treating melasma.[54]

### Flavonoids[12]

Flavonoids are naturally occurring polyphenolic compounds that have well-known anti-inflammatory, antioxidant, antiviral, and anticarcinogenic properties. Many plant-derived flavonoid compounds have hypopigmentary effects and their roles are still under investigation. These include *catechin* conjugated with *gallic acid* (from green tea leaves), *ellagic acid* (from green tea, strawberry, eucalyptus etc), and *aloesin* (from aloe tree).

Other agents known to affect melanin pigmentation and sometimes used in formulations are *N-acetyl glucosamine*, *thiotic acid* (alpha-lipoic acid), *gentisic acid*, *soybean extract,* and *paper mulberry extract.*

## Combination Treatment

Melanogenesis is a complex, multi-stage process in which precursor molecules are acted upon by the enzyme tyrosinase to produce the complex biopolymer named melanin in specific organelles called melanosomes. Melanized melanosomes are then transferred from melanocytes to the keratinocytes, eventually producing the visually apparent skin color. Various topical agents act on different stages of this process, thus providing a rationale for combinations of agents for better therapeutic effect. In addition to having a synergistic effect, a particular drug may abrogate untoward effects of another drug formulated in the same vehicle. For example, topical steroids may reduce the irritant effects of HQ or retinoids. On the other hand, retinoids may prevent steroid-induced cutaneous atrophy. Combinations of hypomelanotic agents with sunscreens are also available in the market.

Various combinations of different topical agents have been studied and many are marketed by pharmaceutical companies. Hydroquinone is generally the main component of formulations. It is combined with drugs like glycolic acid, azelaic acid, kojic acid, retinoic acid, or corticosteroids. In addition, arbitrary mixtures of various other demelanizing agents are marketed although efficacy and safety have not been established by controlled clinical trials for most of them.

However, the most extensively studied and widely used combination is the so-called ‘triple combination’, a formulation containing HQ, retinoic acid, and corticosteroids. First proposed by Kligman and Willis,[55] the original combination utilized 5% HQ, 0.1% tretinoin, and 0.1% dexamethasone and was found to be effective in the treatment of melasma, ephelides, and postinflammatory hyperpigmentation. Concerned about the irritant potential of this combination for its high tretinoin concentration, modifications of the theme have been tried (HQ 4%, tretinoin 0.05%, and fluocinolone acetonide 0.01%) and have been found to be highly effective in long-term clinical studies.[34] It has been suggested that first-line therapy for melasma should consist of effective topical therapies, mainly in the form of triple combinations, and only when triple combinations are unavailable or when patients have hypersensitivity to them, should dual ingredients or single agents be considered.[34]

## Treatment During Pregnancy[23]

Melasma is more resistant to treatment during pregnancy because of the persistent hormonal trigger for development of the disease. For this reason, treatment for melasma is routinely deferred until after delivery. Moreover, treatment may be unnecessary because melasma in pregnancy may be a transient affair; removal of the hormonal trigger after parturition may result in significant improvement.

## Conclusion

Management of melasma can be challenging and requires long-term treatment with topical agents. The results are often unsatisfactory and topical agents may sometimes cause significant adverse reactions. Hydroquinone has remained the gold standard of topical treatment but concerns regarding its side effects remain. A triple combination of hydroquinone, retinoic acid, and corticosteroids has been suggested to be the first-line topical treatment for this pigmentary disorder. Many new agents that inhibit melanogenesis have been developed. Although *in vivo* and *in vitro* experimental studies have suggested their potential role in the management of melasma, controlled clinical trials are mostly lacking and are urgently needed in the future.
